# Optimized ventilation power to avoid VILI

**DOI:** 10.1186/s40560-023-00706-y

**Published:** 2023-11-20

**Authors:** Lauren T. Thornton, John J. Marini

**Affiliations:** Department of Pulmonary and Critical Care Medicine, University of Minnesota, Minneapolis/St Paul, MN USA

**Keywords:** ARDS, VILI, Mechanical ventilation, Mechanical power, Lung protection

## Abstract

The effort to minimize VILI risk must be multi-pronged. The need to adequately ventilate, a key determinant of hazardous power, is reduced by judicious permissive hypercapnia, reduction of innate oxygen demand, and by prone body positioning that promotes both efficient pulmonary gas exchange and homogenous distributions of local stress. Modifiable ventilator-related determinants of lung protection include reductions of tidal volume, plateau pressure, driving pressure, PEEP, inspiratory flow amplitude and profile (using longer inspiration to expiration ratios), and ventilation frequency. Underappreciated conditional cofactors of importance to modulate the impact of local specific power may include lower vascular pressures and blood flows. Employed together, these measures modulate ventilation power with the intent to avoid VILI while achieving clinically acceptable targets for pulmonary gas exchange.

## Causes of ventilation induced lung injury

Three interacting mechanical factors determine the risk of ventilator induced lung injury (VILI). These include (1) achieving inspiratory pressure that exceeds a threshold for excessive strain; (2) applying dangerous stretching forces at a rate sufficient to overcome innate adaptive responses; (3) sustaining an injurious pattern of above-threshold cycles for sufficient time (duration). Since publication in the year 2000 of the landmark study by the ARDSnet that compared outcomes using tidal volumes of 6 versus 12 ml/kg [1], improved understanding of the VILI stimulus has helped to inform an approach that better serves a lung protective strategy. Through two decades of effort we have progressed from prioritizing lower plateau pressure [2], to fully ‘opening the lung’ using aggressive recruitment and higher positive end expiratory pressure (PEEP) [3], to our current emphasis on restricting the driving pressure—the difference between plateau pressure and PEEP measured during passive inflation [4]. Each of these approaches have added value to our current understanding of the root mechanical cause of VILI, but all restrict their focus to the individual inflation cycle.

In recent years attention has been drawn to incorporating the frequency with which damaging energy may be applied by each of those tidal cycles [5]. At the present time, the driving pressure of the passive inflation cycle, the ratio of tidal volume to respiratory system compliance, has been identified as the most influential ventilatory determinant of adverse outcomes [4, 6]. But it is highly likely that controlling measurable driving pressures without simultaneous attention to the end-inspiratory alveolar pressures achieved, the frequency of their application, tidal volume, and such non-mechanical co-contributors to VILI as disease stage, innate tissue vulnerability, body positioning, hemodynamics, and diverse alveolar microenvironments will ignore important aspects of a maximally lung protective ventilation strategy.

**Fig. 1:**
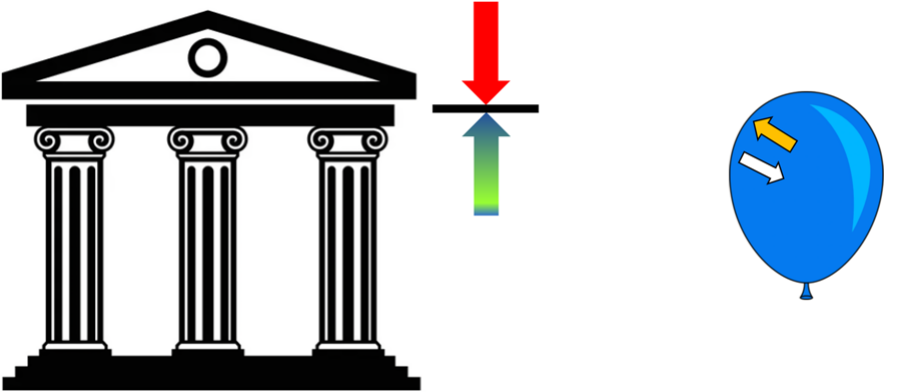
Examples of opposing forces in equilibrium. With forces in balance and without movement, pressures at the extremes of the tidal cycle, *P*_Plateau_ and PEEP, do not alone cause damage

Despite strong correlation of driving pressures that exceed 15 cm of water with mortality across a broadly defined population characterized as having acute respiratory distress syndrome (ARDS) [4], such correlation does not imply causation. Indeed, because both components of driving pressure (the PEEP and plateau pressure) are measured at lung rest, it is reasonable to ask whether either of these static pressures—or their difference—can logically cause tissue damage (Fig. 1). High alveolar pressures and pressure differences may be necessary to induce VILI, but of themselves are not sufficient conditions if detached from the flows and volumes they deliver. Considered alone, static pressures imply a balance of forces and do not fully characterize the energy needed to inflict damage. Attention is more logically directed toward the active (dynamic) process that expends the energy needed to overstretch lung tissue or cause repeated cycles of tidal opening and closure of small airways (a form of VILI termed ‘atelectrauma’ [7]).

## Pressure and local lung stress

Static pressures measured at the airway opening, such as the plateau pressure and PEEP, reflect average values whose local distending forces are co-determined by the specific microenvironment that surrounds the individual lung unit. When estimated as the difference between airspace and regional pleural pressure, these transpulmonary pressures vary from site -to-site within the lung, with non-dependent units distended more at end-expiration than those that are gravitationally dependent [8]. In the supine body orientation the gradient of transpulmonary pressure is greater than when prone, in which positional mechanical forces that locally distend lung units are distributed more equally [9]. The dimension, local flexibility, and strain of individual units within the reduced ventilated lung tissue of ARDS (the functional ‘baby lung’) vary with their locale, as well as with the stage of the injury process [10].

**Fig. 2:**
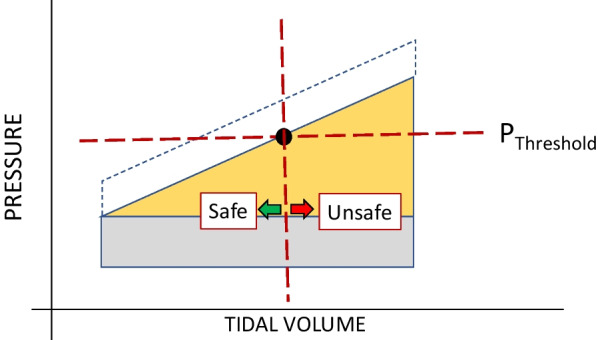
Elastic pressure vs. inflation volume. The area enclosed beyond the threshold pressure (*P*_Threshold_) represents the ‘unsafe’ tidal elastic energy applied under high stress that holds greater damaging potential

**Fig. 3:**
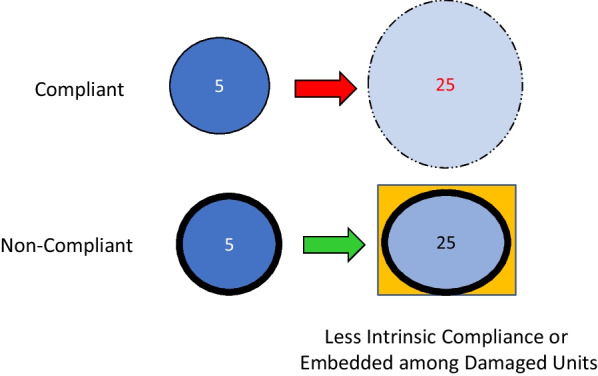
Dependence of strain on lung unit compliance. A high driving pressure of 20 cmH_2_O (25 – 5 cmH_2_O) may overstretch a compliant unit but does not damage one with intrinsically low compliance or one embedded within and buttressed by surrounding tissue that prevents its intolerable expansion

Repeated application of cycles that overstrain the cellular membrane requires a volume change driven by pressure. The stretch (strain) of inflation, linked presumably to its potential for delivering damaging energy, is a function of the total transpulmonary pressure (due to PEEP plus the airway pressure above PEEP needed to deliver the added tidal volume) and the simultaneous volume change that occurs (Fig. 2). For the entire lung, the tidal compliance that determines its average stretch is calculated as the ratio of the volume change (tidal volume) and the driving pressure. ‘Overstretch’ of an alveolus, also designated as ‘overdistention’ or ‘hyperinflation’, occurs when the applied driving pressure and its associated volume increment exceeds its tolerable strain limit. This threshold of injury hazard is raised by tissue strength and lowered by fragility (vulnerability). Note that fragile lung units that are surrounded and buttressed by consolidated tissue also may be relatively spared intolerable tidal expansion (Fig. 3). As discussed later, there are several amplifiers of stress that apply to each transpulmonary pressure within the mechanically heterogeneous lung.

## Fundamental properties of energy and power

**Fig. 4:**
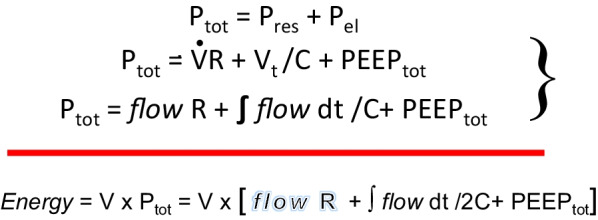
Components of tidal energy. Total pressure is the sum of its flow resistive, driving, and end-expiratory components. When coupled to volume increments, these individual pressure categories contribute to total inflation energy (*P*_tot_ = Total pressure, *P*_res_ = flow resistive pressure, *P*_el_ = conserved (‘elastic’) pressure, *R* = flow resistance, *V*_t_ = tidal volume, *C* = tidal compliance, PEEP_tot_ = PEEP + auto-PEEP, *V* = Volume above end-expiratory baseline value)

By the first law of thermodynamics, the energy in a ‘closed’ system can neither be created or destroyed—only transformed [11]. Mechanical energy can take the form of potential energy or kinetic energy that can, in turn, expand, deform, damage, or dissipate as heat. Static pressures (e.g., PEEP and plateau pressure) are key components of potential energy storage that when built or released drive alveolar volume changes. In building toward the inspiratory plateau pressure, any volume increment resulting in excessive stretch may impose damage. The total inflation pressure that powers the tidal volume change is comprised of three elements: (1) the flow resistive pressure; (2) the incremental component (related to driving pressure); and (3) positive end expiratory pressure (total PEEP) [12]. Each pressure component contributes to the energy of its corresponding sector. Those three sectors comprise the total inflation energy (Fig. 4). The instantaneous product of pressure and flow is the moment-by-moment (intracycle) power applied to these energy sectors [13].

**Fig. 5:**
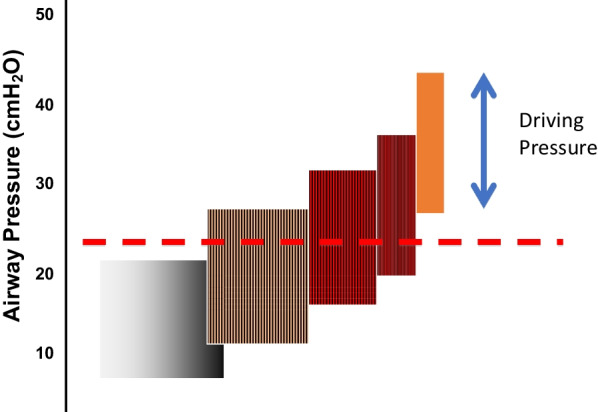
Influence of raising PEEP on recruitment and strain of stress-focused junctional alveoli in zones where open and closed units are contiguous. With the same driving pressure applied, increasing PEEP reduces the number of high-risk interfaces but increases the stress applied at ‘above- threshold’ pressures to those interfaces that remain unrecruited (dashed horizontal red line = threshold pressure)

Flow, the volume change per unit time, can be defined on any time scale. In the clinical literature it has recently become customary to use the term ‘power’ to describe the product of frequency per minute and total inflation energy per cycle [14]. Actually, this descriptor is better thought of as a cumulative measure of the monotonous and interrupted energies of multiple inflation cycles. As a single variable integrating information from all machine settings shown individually to influence ventilator induced lung injury [15], the current clinical term ‘power’ has drawn increased attention as a modifiable and observable parameter with which to regulate VILI risk [16]. Yet, rather than the current practice of basing ‘power’ on the entirety of inflation energy, it seems more physiologically justifiable to recognize that an elastic pressure threshold must first be crossed before additional energy from that inflation cycle overstretches. Consequently, not total power (energy per minute) but ‘damaging power’ may be a more desirable and precise indicator of the VILI hazard [17]. Assuming that to be the case, it also stands to reason that the addition of PEEP brings any applied driving pressure closer to crossing the threshold of overstretch and injury risk for some units of the injured lung (Fig. 5). On the other hand, PEEP exerts a beneficial action to the extent it recruits new lung units to share the stress and energy load, as discussed later. Because the injured lung is mechanically heterogeneous, both competing processes—one increasing risk and one decreasing risk, are in play whenever PEEP is adjusted [8, 18].

## Mechanical factors that amplify stress

Four important stress amplifiers (stress risers) are in play in the mechanically heterogeneous lung of ARDS. These include structural asymmetries, flow dependent viscoelastic drag, progressive and sequential loading due to structural unit dropout, and reduced ventilating capacity of the aerated ‘baby lung’ of ARDS.

### Reduced ventilating capacity

**Fig. 6:**
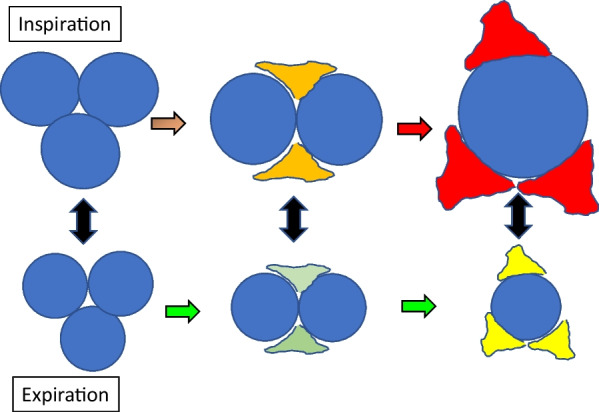
Impact of reduced alveolar numbers on end-tidal inflation. As the number of open units declines, each absorbs more energy and distends further at end inspiration, as it must accommodate a greater share of the tidal volume. Note that end-expiratory volumes remain unchanged

Perhaps the most important of the four key amplifiers of the stress applied by alveolar pressure is the reduced size of the aerated baby lung. To accomplish alveolar ventilation, a smaller lung must stretch to a greater dimension per cycle and/or do so at a greater rate. Lower capacity means that energy needed to deliver the tidal volume distributes to a smaller number of units, increasing their individual (‘specific’) power loads (Fig. 6). Once a damaging process is underway, therefore, the potential exists for a rapidly progressing process of force and power amplification, a condition that has been described as a ‘VILI vortex’ of advancing injury [19].

### Structural unit dropout

**Fig. 7:**
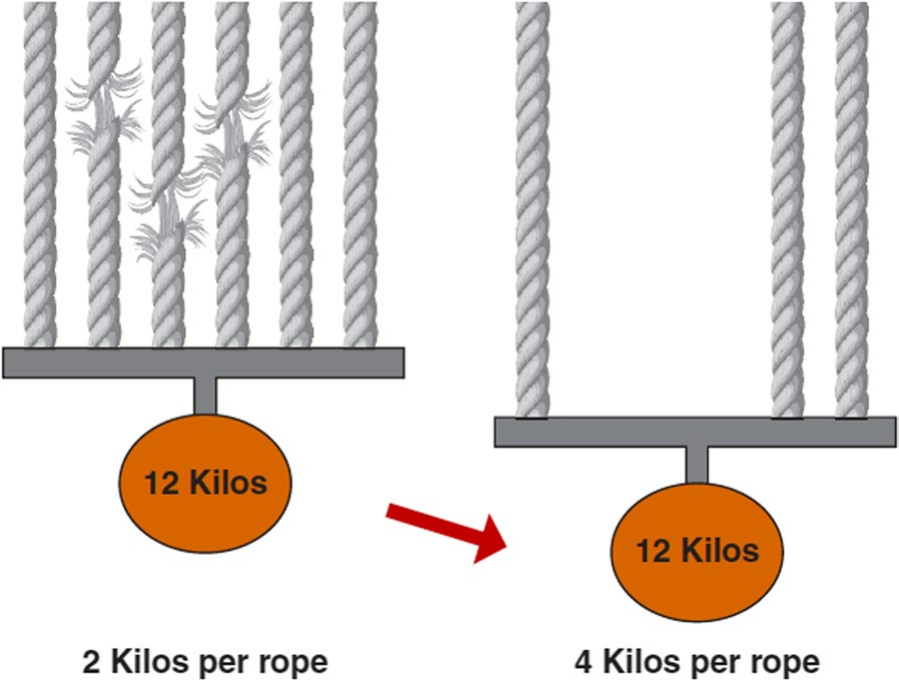
Effect of dropout on strain per element in a shared supporting network of ropes (analogous to biologic microfibrils). As weaker stress-bearing elements break and no longer help support the unchanging load, those remaining intact experience greater stress, strain and mechanical energy (Kilos = kilogram weights)

The supporting interstitial matrix that bears a substantial portion of the stress load of inflation has a fibrillar structure whose individual elements share that burden of expansion [20]. Sequential dropout of weaker stress bearing elements requires the remaining intact fibrils to take up additional stresses and strain more during inflation (Fig. 7). Thus, once beyond the injury threshold, the stretching forces applied to intact units may increase progressively. This augmented stress may, in turn, overtax the next weakest fibrils of the bundle, encouraging an erosive process that shrinks the ‘baby lung’ [19].

### Structural asymmetry

Stresses are amplified at the junctions of easily expanding units with those that are reluctant or impossible to inflate. Such interfaces experience amplified tension [21, 22], and these tend to prevail at the low lung unit volumes of gravitationally dependent locations. Although they occur in both supine and prone positions, such high-risk interfaces form more extensively in the former, perhaps accounting in large part for the lung protective benefit of proning that has been directly demonstrated experimentally [23] and inferred from the results of large clinical trials [24].

### Viscoelastic drag

Rapid expansion accentuates the stress applied by a given pressure to tissues that are reluctant to expand because of increased viscoelastance [21, 22]. During active inflation, this ‘drag’ is a dynamic amplifying force that compliments heterogeneous geometrical stress focusing. Indeed, reducing peak flow exposure has been demonstrated to attenuate VILI-related lung damage in large animals [25].

#### Influence of flow

**Fig. 8:**
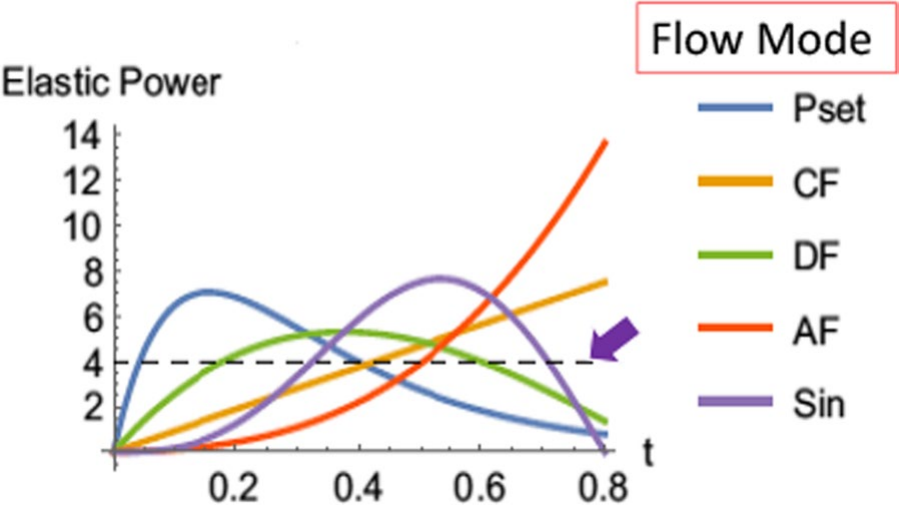
Theoretical influence of inspiratory flow profile on instantaneous elastic power. The total energy required to deliver a given tidal volume is the same for all profiles. However, different flow patterns apply their power intensity at different times. Consequently, energy delivery may crest over a damaging threshold earlier or later in the inflation period (Pset = Pressure control, CF = Constant flow, DF = Linearly decelerating flow, AF = Accelerating flow, Sin = Sinusoidal flow)

An underemphasized potential contributor to local force amplification owes to the amplitude and profile of the flow pattern utilized in tidal volume delivery [13]. Apart from the stretch amplification via viscoelastic drag, flow amplitude and profile settings drive specific flows through the reduced number of channels that supply the baby lung. These abnormally higher local flow rates dissipate energy and of themselves hold injury potential. Moreover, the instantaneous power applied to the lung distributes in accordance with local tissue flexibility and time constant [26]. All flow profiles ultimately deliver the same total of elastic energy to the entire lung by the end of the inflation cycle. However, decelerating flow profiles deliver a higher percentage of that total energy relatively early in the inflation cycle, whereas constant flow delivers the same energy load more evenly [26] (Fig. 8). In theory, the potential exists for the specific power applied with a decelerating profile to cross the pressure threshold that risks damage to some vulnerable units. Such a difference among flow profiles was demonstrated 20 years ago in a rabbit model of VILI [27]. That important experiment demonstrated greater damage resulting from pressure control than from constant flow delivering the same tidal volume with similar driving pressure, and inspiratory time. Such work is consistent with the notion that constant flow is the preferable waveform for delivering a given tidal volume over a specified inspiratory time to high-risk lung tissues.

## Importance of resting lung volume (FRC) to damaging energy and power

At the bedside, the resting size of the baby lung, the functional residual capacity (FRC), is not routinely measured. However, the risk carried by a given pattern of minute ventilation, driving pressure and PEEP is critically dependent on the capacity of the baby lung into which it is applied. It is generally believed that the lung units that remain open in the acutely injured lung of ARDS function relatively normally [10, 28]. Whatever the truth of this perception, the aerated baby lungs of ARDS are inherently inefficient in achieving sufficient alveolar ventilation to keep pace with the body's needs; fewer lung units must therefore work harder than when healthy. For reasons already discussed, power concentrates and flow velocities increase within lungs of reduced capacity, unlike static airway pressures, which distribute uniformly. Such increases of ‘specific power’ raise the likelihood of crossing local elastic pressure thresholds for damage.

## Role of pulmonary pressure and perfusion

**Fig. 9:**
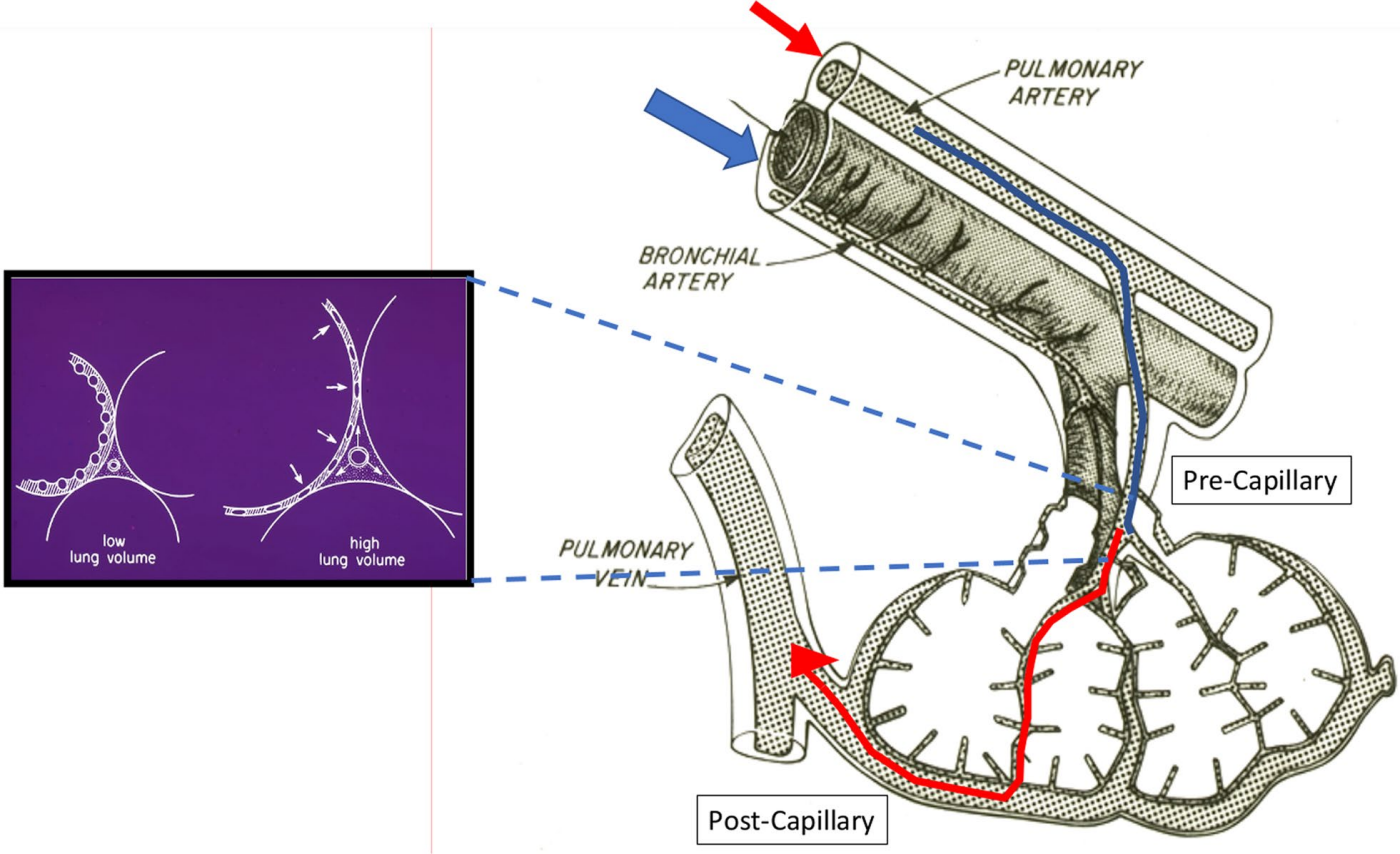
Vascular effects of alveolar inflation. During expansion, high stresses are applied to the junctions of capillary and interstitial micro vessels, as the former are compressed and the latter dilated in that process. Moreover, greater rates of blood flow through the reduced bed of the ‘baby lung ‘amplify endothelial shear and locally dissipate vascular energy

Another important but underappreciated aspect of VILI risk is attributable to the high blood flows coursing through the baby lung. Note that the entire cardiac output must be accommodated by the lung, and while its aerated (‘baby lung’) portion might be as little as 20% of its healthy counterpart, *non-shunted* vascular flow usually exceeds 70% of cardiac output [28, 29]. Heightened specific perfusion through the baby lung, therefore, may impose intolerable stress across its already abnormal vascular endothelium. Indeed, experimental data show convincingly that for the same applied airway pressures and airflows, the gradient of trans alveolar vascular pressure is a critical determinant of observed hemorrhagic changes [30, 31]. Such adverse effects are accentuated by alveolar overdistension and by increasing cardiac output (Fig. 9). Both blood flow and end-tidal overdistention are increased in the baby lung. It stands to reason that reducing total body oxygen demand, avoiding unnecessary lung unit distension due to high PEEP levels and opting for prone body positioning to improve oxygen exchange and recruit blood vessels [32] would reduce trans alveolar pressure gradients, endothelial stretch and dissipated *vascular* energy due to excessive perfusion.

## CARDS and VILI risk

Recent experience with the ARDS caused by COVID-19 pneumonia (CARDS) has provided insights into the value of several lung protective measures. Prone positioning proved a highly useful intervention during the pandemic, not only to boost oxygenation efficiency, but also to delay or avert the need for intubation [33]. Although its mechanism(s) of action in C-ARDS may be debatable, prone positioning clearly reduced the heterogeneity of mechanical forces and redistributed both ventilation and blood flow beneficially. Improved V/Q matching by prone positioning of spontaneously breathing patients helped avoid the need for high PEEP application to distend the lung. Indeed, in these initially gas filled and compliant lungs, customarily applied levels of PEEP often had unexpected adverse physiological effects [34]. In those patients who failed to improve, respiratory system compliance, which in the early stage was often relatively normal despite hypoxemia, markedly deteriorated over time as did the ventilatory ratio, indicating worsened ventilation efficiency [35]. Oxygenation on the other hand, tended to remain more or less unchanged throughout the entire disease course. Such observations indicate that mechanisms were at work that prominently involved the vasculature as well as airspaces, with pathology and imaging differing from those patterns encountered with usual forms of ARDS [34–36].

## Paradoxical improvement of compliance and lung stress by chest wall modification

**Fig. 10:**
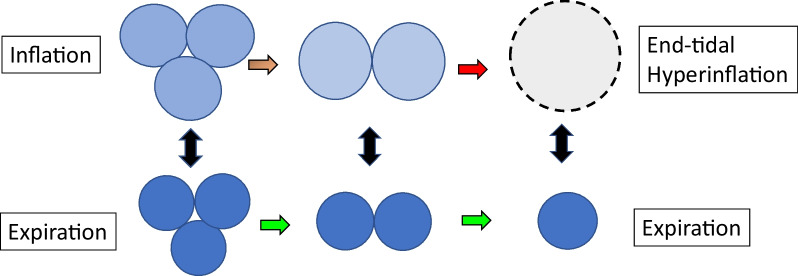
End-tidal hyperinflation in units of very small ‘baby lungs’ of ARDS. Delivery of the tidal volume forces overstretching to accommodate added volume, even as end-expiratory dimension remains unaffected

As respiratory system mechanics worsened in CARDS, aerated capacity markedly declined, indicating falling numbers of well-functioning gas exchange units. Clinician-set tidal volumes then distributed into fewer lung alveoli, amplifying their specific power exposures. This heightened burden of ventilatory energy resulted in airspace overdistension at end-inflation, despite a lack of significant dynamic hyperinflation at end-expiration (auto-PEEP) [37] (Fig. 10). This combination of factors gave rise to very low measured compliance, elevated plateau pressures, and a relatively high incidence of lung unit rupture and barotrauma [38]. Detection of end inspiratory hyperinflation was elicited by observing the so-called ‘paradoxical’ effects of chest wall compression. Contrary to usual behaviors and expectations, external pressure improved (rather than worsened) respiratory compliance, driving pressure, and plateau pressure [39, 40]. Therapeutic measures to avoid end-tidal hyperinflation are characterized by reduction of the end-inspiratory volume. These included using lower tidal volume, lower PEEP, more horizontal body positioning, and perhaps sustained chest wall compression by abdominal binder [41–43]. Notably, the prone orientation combines horizontal positioning with regional compression of the ribcage and abdomen [44, 45]. Apart from using these measures to reduce the stress and strain of individual tidal cycles, damaging power exposure can be addressed by reducing the need for ventilation (adopting a target of modest permissive hypercapnia), lowering the body’s demand for oxygen, and in extreme cases initiating extracorporeal carbon dioxide removal or oxygenation. Such interventions not only limit the energy and blood flows delivered per inflation cycle but also allow reduced frequency of their application.

## Summary

The main determinants of ventilator induced lung injury are intolerable energy delivered per cycle, the frequency of tidal cycling, and the duration of ventilation. Hazardous energy per cycle delivers a tidal volume that crosses an elastic pressure threshold and exceeds the ‘baby lung’s capacity to accept it without overstretching. The pace and profile of flow delivery may also be important factors determining risk in the highly vulnerable lung. That vulnerability is determined not only by the lung’s endogenous inflammatory state, but by also by its perfusion and disease stage. For a given transpulmonary pressure, stress amplifiers include extent of mechanical heterogeneity, reduced size of the baby lung, and progressive drop out of lung units—a process that accentuates the load on the remaining stress-bearing elements that support alveolar tissues.

The effort to avoid VILI risk, therefore, must be multi-pronged. The need to ventilate, a key determinant of hazardous power, is reduced by judicious permissive hypercapnia, reduction of innate oxygen demand, and prone body positioning that promotes efficient pulmonary gas exchange and more equal distributions of local stress. Modifiable ventilator-related determinants of lung protection include reductions of tidal volume, plateau pressure, driving pressure, PEEP, inspiratory flow amplitude and profile (using longer inspiration to expiration ratios), and ventilation frequency. Underappreciated conditional cofactors of importance to modulate the impact of local specific power may include lower vascular pressures and blood flows. Employed together, these measures modulate ventilation power with the intent to avoid VILI while achieving targets for pulmonary gas exchange.

## Data Availability

Not applicable.
